# Active integration of glutamatergic input to the inferior olive generates bidirectional postsynaptic potentials

**DOI:** 10.1113/JP273424

**Published:** 2016-11-29

**Authors:** Derek L. F. Garden, Arianna Rinaldi, Matthew F. Nolan

**Affiliations:** ^1^ Centre for Integrative PhysiologyUniversity of EdinburghEdinburghEH8 9XDUK

**Keywords:** inferior olive, ion channel, synaptic integration

## Abstract

**Key points:**

- We establish experimental preparations for optogenetic investigation of glutamatergic input to the inferior olive.
- Neurones in the principal olivary nucleus receive monosynaptic extra‐somatic glutamatergic input from the neocortex.
- Glutamatergic inputs to neurones in the inferior olive generate bidirectional postsynaptic potentials (PSPs), with a fast excitatory component followed by a slower inhibitory component.
- Small conductance calcium‐activated potassium (SK) channels are required for the slow inhibitory component of glutamatergic PSPs and oppose temporal summation of inputs at intervals ≤ 20 ms.
- Active integration of synaptic input within the inferior olive may play a central role in control of olivo‐cerebellar climbing fibre signals.

**Abstract:**

The inferior olive plays a critical role in motor coordination and learning by integrating diverse afferent signals to generate climbing fibre inputs to the cerebellar cortex. While it is well established that climbing fibre signals are important for motor coordination, the mechanisms by which neurones in the inferior olive integrate synaptic inputs and the roles of particular ion channels are unclear. Here, we test the hypothesis that neurones in the inferior olive actively integrate glutamatergic synaptic inputs. We demonstrate that optogenetically activated long‐range synaptic inputs to the inferior olive, including projections from the motor cortex, generate rapid excitatory potentials followed by slower inhibitory potentials. Synaptic projections from the motor cortex preferentially target the principal olivary nucleus. We show that inhibitory and excitatory components of the bidirectional synaptic potentials are dependent upon AMPA (GluA) receptors, are GABA_A_ independent, and originate from the same presynaptic axons. Consistent with models that predict active integration of synaptic inputs by inferior olive neurones, we find that the inhibitory component is reduced by blocking large conductance calcium‐activated potassium channels with iberiotoxin, and is abolished by blocking small conductance calcium‐activated potassium channels with apamin. Summation of excitatory components of synaptic responses to inputs at intervals ≤ 20 ms is increased by apamin, suggesting a role for the inhibitory component of glutamatergic responses in temporal integration. Our results indicate that neurones in the inferior olive implement novel rules for synaptic integration and suggest new principles for the contribution of inferior olive neurones to coordinated motor behaviours.

## Key points

- We establish experimental preparations for optogenetic investigation of glutamatergic input to the inferior olive.
- Neurones in the principal olivary nucleus receive monosynaptic extra‐somatic glutamatergic input from the neocortex.
- Glutamatergic inputs to neurones in the inferior olive generate bidirectional postsynaptic potentials (PSPs), with a fast excitatory component followed by a slower inhibitory component.
- Small conductance calcium‐activated potassium (SK) channels are required for the slow inhibitory component of glutamatergic PSPs and oppose temporal summation of inputs at intervals ≤ 20 ms.
- Active integration of synaptic input within the inferior olive may play a central role in control of olivo‐cerebellar climbing fibre signals.

**Abbreviations**
AAV: adeno‐associated virus
ChR2: channelrhodopsin 2
GluA: AMPA receptor
IO: inferior olive
PON: principal olivary nucleus
PSP: postsynaptic potential

## Introduction

Coordination and timing of movement rely on integration of motor and sensory signals by the olivo‐cerebellar system (Apps & Garwicz, 2005; De Zeeuw *et al*. 2011). Within this system the climbing fibre output from the inferior olive (IO) organizes neuronal activity and plasticity in the cerebellar cortex (Mathy *et al*. 2009; De Zeeuw *et al*. 2011; Badura *et al*. 2013). Because climbing fibre activity is essential to normal cerebellar function and as neurones in the IO have unusual oscillatory membrane potential dynamics, the computations that IO neurones carry out are of particular interest (De Zeeuw *et al*. 1998; Llinás, 2009). Sinusoidal oscillatory activity, arising from interactions between voltage‐gated ion channels and synchronized within networks of IO neurones connected by gap junctions, has received considerable attention (Llinás & Yarom, 1981; Benardo & Foster, 1986; Bal & McCormick, 1997; Long *et al*. 2002; De Zeeuw *et al*. 2003; Bazzigaluppi *et al*. 2012). In contrast, while the IO is a major site for convergence of synaptic signals (De Zeeuw *et al*. 1998; Apps & Garwicz, 2005), remarkably little is known about how IO networks integrate synaptic inputs to generate climbing fibre outputs.

Because the IO integrates diverse afferent signals (Armstrong, 1974; De Zeeuw *et al*. 1998), the mechanisms that determine its responses to synaptic input are likely to be critical for computations that it carries out. Intracellular recordings from IO neurones in anaesthetized animals reveal excitatory and inhibitory postsynaptic potentials (PSPs) following electrical stimulation of the mesodiencephalic junction (Ruigrok & Voogd, 1995), motor cortex (Crill, 1970) or the cerebellar nuclei (Bazzigaluppi *et al*. 2012). Anatomical evidence suggests that in the cat projections from the motor cortex primarily target the medial accessory nucleus of the IO (Sousa‐Pinto, 1969; Saint‐Cyr, 1983), but the functional properties of these or other long‐range projections are for the most part unclear. Synaptically driven PSPs have also been observed following electrical activation of inputs to the IO in brain slices (Llinás & Yarom, 1981; Bal & McCormick, 1997; Best & Regehr, 2009; Mathy *et al*. 2009; Lefler *et al*. 2014). Fast excitation is presumably mediated by glutamatergic inputs, whereas inhibition from the cerebellar nuclei involves asynchronous release of GABA (Best & Regehr, 2009). Theoretical models suggest that the complex electrical properties of IO neurones may be important for integration of their synaptic inputs (Kistler & De Zeeuw, 2005). These models predict that glutamatergic inputs to IO neurones will trigger bidirectional membrane potential responses, with inhibitory components that require activation of potassium channels. These predictions are yet to be tested experimentally.

To investigate synaptic integration within the IO we established optogenetic methods for selective activation of its long‐range synaptic inputs. We find that glutamatergic postsynaptic potentials (PSPs), including those originating from the neocortex, have excitatory and inhibitory components. We show that synaptic projections from the motor cortex preferentially target the principal olivary nucleus. Analysis of the relationship between inhibitory and excitatory components of glutamatergic PSPs suggests they share a common synaptic origin, with inhibitory components resulting from activation of calcium‐activated potassium channels. The inhibitory components appear to suppress summation of responses to inputs at intervals ≤ 20 ms. Together our results identify functional consequences of activation of long‐range glutamatergic synaptic inputs to the IO and demonstrate a critical role for active membrane conductances in synaptic integration by neurones in the IO.

## Methods

All experiments were carried out under a project licence granted by the UK Home Office and approved by the University of Edinburgh's animal welfare committee. C57BL6 mice, and mice expressing channelrhodopsin 2 (ChR2) under the control of the Thy1‐promoter (Thy1‐ChR2‐YFP line 18, stock number 007612 from The Jackson Laboratory, Bar Harbor, ME, USA) (Arenkiel *et al*. 2007), were housed on a 12 h light/dark cycle (light on 07.30–19.30 h) in standard breeding cages. Experiments used mice of either sex. The median age of mice used was 46 days (range 28–116 days). We did not find any change in measured parameters with age of the mice.

### Electrophysiology

Coronal brainstem slices containing the IO were prepared as follows. Mice were decapitated following isoflurane anaesthesia, their brains rapidly removed and placed in cold (approximately 4°C) modified ACSF of composition (mm): NaCl (86), NaH_2_PO_4_ (1.2), KCl (2.5), NaHCO_3_ (25), glucose (25), CaCl_2_ (0.5), MgCl_2_ (7) and sucrose (75). The brain was placed ventral‐side up and a coronal cut made through the widest part of the brainstem and cerebellum. The cut surface was glued to the stage of a VT1200S sectioning system (Leica Microsystems UK Ltd, Milton Keynes, UK), with the caudal part of the brain facing upwards. Coronal sections of thickness 200 μm, to enable visualization of neurones for recording, were cut submerged under cold (approximately 4°C) modified ACSF. Slices were transferred to a storage container filled with standard ACSF of composition (mm): NaCl (124), NaH_2_PO_4_ (1.2), KCl (2.5), NaHCO_3_ (25), glucose (20), CaCl_2_ (2) and MgCl_2_ (1). Slices were maintained at 33–35°C for 10–20 min and then allowed to cool to room temperature (20–24°C). Approximately 20% (*n* = 10/51 neurones, *N* = 10/43 mice, mean frequency: 5.8 ± 0.3 Hz, *n* = 10) of recorded neurones showed periods of spontaneous oscillatory activity. To avoid contamination of responses by this activity we did not stimulate synaptic input during these periods.

For recording, slices were transferred to a submerged chamber and neurones in the IO were visually identified under infrared illumination with differential interference contrast (DIC) optics. Whole‐cell recordings were obtained at 35–37°C from the soma of IO neurones using electrodes with resistance 2–5 MΩ when filled with intracellular solution comprising (mm): potassium methyl sulfate (130), KCl (10), Hepes (10), MgCl_2_ (2), EGTA (0.1), Na_2_ATP (4), Na_2_GTP (0.3) and phosphocreatine (10). Recordings were made in current‐clamp configuration with series resistances < 40 MΩ. Appropriate bridge and electrode capacitance compensations were applied. Membrane potential was filtered at 5–10 KHz and sampled at 20–50 KHz.

For optogenetic activation of ChR2‐expressing axons a light‐emitting diode (LED) attached to the epifluorescence port of the microscope delivered 3 ms‐duration pulses of 480 nm wavelength light. Activation of the LED was controlled by an analog voltage output from a data acquisition board. Light stimulation intensity was quantified as the power of the light measured by a light meter (Thorlabs, Ely, UK).

All chemicals were purchased from Sigma (St Louis, MO, USA) with the exception of apamin, iberiotoxin, 2,3‐dihydroxy‐6‐nitro‐7‐sulfamoyl‐benzo[f]quinoxaline‐2,3‐dione (NBQX), d‐2‐amino‐5‐phosphonovaleric acid (d‐AP5) and picrotoxin from Abcam biochemicals (Cambridge, UK). Drugs were made fresh daily from frozen stocks concentrated 1000‐fold.

### Viral injections

Mice were deeply anaesthetized with isofluorane and then placed in a stereotaxic frame. A cut was made to expose the skull, holes were drilled bilaterally above areas of neocortex containing M1 and M2 (1.2–1.4 ML, 1.0–1.5 AP from bregma), and the underlying dura were carefully removed. A pipette was then inserted at a depth of 1 mm from the pial surface and 500 nl of adeno‐associated virus (AAV) (pACAGW‐ChR2‐Venus, Vector Biolabs, Malvern, PA, USA or CBA‐synaptophysin‐eGFP, Groh *et al*. 2008) was injected over the course of 5 min. Pipettes were left for 5 min post‐injection before removal. All mice were given a subcutaneous injection of Vetergesic (Henry Schein, UK) prior to surgery and access to vetergesic in jelly form during recovery. Mice were left for at least 3 weeks after surgery before being used for electrophysiological recordings or anatomical analysis. All mice showed Venus or enhanced green fluorescent protein (eGFP) expression in M1, including layer 5 projection neurones (*N* = 13/13 and 5/5, respectively). Some mice expressed ChR2 in M2 and in deep layers of Cg1. Because labelling in the IO was similar in these mice (*N* = 9 and 2, respectively), we pooled these data with data from mice in which expression was restricted to M1.

### Histology

Analysis of the expression pattern of Venus or eGFP was as described previously (Sürmeli *et al*. 2015). Briefly, anaesthetized mice were perfused with cold PBS followed by 4% cold paraformaldehyde (PFA) or formalin and the brain removed. After fixation brains were washed in phosphate buffer and sectioned. Sections were counter‐stained with NeuroTrace 640/660 (Thermo Fisher Scientific, Renfrew, UK, 1:1000) or with antibodies against NeuN (MAB377, Millipore, Watford, UK) and mounted for imaging.

### Data analysis

Electrophysiological data were analysed in IGOR Pro (Wavemetrics, Lake Oswego, OR, USA) using Neuromatic (http://www.neuromatic.thinkrandom.com/) and custom‐written routines, or using AxoGraph (axographx.com). Imaging and analysis of axons and synaptic terminals were carried out as described previously (Sürmeli *et al*. 2015). For quantification of fluorescence intensity a region of interest (ROI) was drawn around each nucleus using Fiji (https://fiji.sc/) (Schindelin *et al*. 2012). The total signal was measured, normalized to the area of the ROI and then the normalized signal measured from a region devoid of terminal labelling was subtracted. Statistical analysis was carried out using IGOR Pro, Excel (Microsoft), or R (www.R-project.org). Numbers of mice used (*N*) and numbers of cells recorded (*n*) are reported for each analysis. All statistical analyses use *n*. Mean values are ± standard error of the mean (SEM). Statistical significance was tested with linear regression, Student's *t* test or two‐way repeated measures ANOVA followed by Bonferroni–Holm *post hoc* tests where appropriate. Adjusted *R* ^2^ values are stated for results of linear regression. Input resistance was calculated from the steady‐state voltage response to injected −80 pA current steps.

## Results

**Figure 1:**
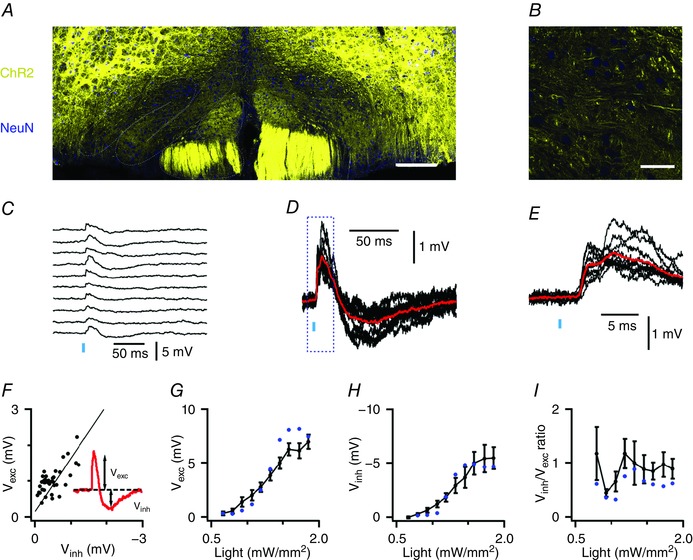
Long‐range synaptic inputs generate bidirectional responses in IO neurones *A*, coronal brainstem section from a Thy1‐ChR2‐YFP mouse. YFP‐positive cell bodies (yellow) are primarily outside of the IO (dashed lines). Scale bar 100 μm. *B*, expanded region from *A*. Scale bar 50 μm. *C*, voltage responses of an IO neurone from a Thy1‐ChR2‐YFP mouse to 10 consecutive light stimuli (vertical bar, intensity = 0.92 mW mm^−2^). *D* and *E*, traces from *C* overlaid at a higher gain (*D*) and at a faster time scale (*E*). Average responses are in red. *F*, the amplitude of each excitatory (*V*_exc_) component as a function of the inhibitory (*V*_inh_) component when inputs are activated with a fixed stimulus intensity (*R* ^2^ = 0.48, *P* = 3.6 × 10^−7^). Data are from the cell in *C–E*. The line indicates linear regression. *G–I*, mean *V*_exc_ (*G*) and *V*_inh_ (*H*), and the ratio *V*_inh_/*V*_exc_ (*I*) are plotted as a function of stimulus intensity (*n* = 6 cells). Blue circles are data from the neurone in *C–E*.

We established two preparations for selective activation of long‐range inputs to IO neurones (Figs 1 and 3). In a first approach, we used mice that express channelrhodopsin 2 (ChR2) and yellow fluorescent protein (YFP) under control of the Thy1 promoter (Thy1‐ChR2‐YFP mice) (Arenkiel *et al*. 2007). In a second approach we expressed ChR2 from an AAV vector injected into the motor cortex.

### Activation of long‐range synaptic inputs to the IO generates bidirectional synaptic responses

In Thy1‐ChR2‐YFP mice ChR2 is expressed by several neuronal populations believed to project to the IO, including neurones in the neocortex, midbrain and spinal cord, but not neurones in the cerebellar nuclei (Arenkiel *et al*. 2007). In these mice labelled axons from neurones expressing ChR2 are found in all nuclei of the IO, whereas labelling is absent from cell bodies, indicating that ChR2 is found exclusively at presynaptic locations (Fig. 1 *A* and *B*). To investigate the functional properties of the projections from ChR2‐expressing neurones we made patch‐clamp recordings from IO principal neurones identified by their large soma and characteristic action potential afterdepolarization (Llinás & Yarom, 1981). Light stimulation consistently evoked bidirectional postsynaptic potentials (PSPs) during which an initial depolarization was followed by a longer hyperpolarization (Fig. 1 *C–E*). Closer examination of the initial depolarization revealed a first phase with a latency of 2.5 ± 0.2 ms (*n* = 7, *N* = 7) that plateaus within a further ∼5 ms and is followed by a second smaller depolarization (Fig. 1 *E*). The later inhibitory response peaked at 64.9 ± 6.5 ms after stimulation (*n* = 7, *N* = 7). Similar bidirectional responses were observed in all neurones tested (*n* = 55/55), indicating that rapid excitation followed by slower inhibition is a general feature of IO neurone responses to long‐range synaptic input.

To begin to understand the relationship between the excitatory and inhibitory components of the PSP, we asked if each component is the result of activation by different populations of presynaptic axons. We reasoned that if the two components are generated independently, then, when a constant number of presynaptic axons is activated, the trial to trial variability in their amplitudes should be independent of one another. We tested this for six cells (*N* = 5 mice) by examining 20–50 consecutive responses to stimulation at a fixed light intensity. In contrast to our prediction for responses generated by independent mechanisms, we found a strong correlation between the amplitudes of the excitatory and inhibitory components (Fig. 1 *F*) (mean *R* ^2^ = 0.48 ± 0.06, range *R* ^2^ = 0.39–0.78, *P* = 1.2 × 10^−3^–1.43 × 10^−7^). Thus, excitatory and inhibitory components of the PSP may result from a common synaptic pathway.

We next investigated the consequences of varying the number of activated presynaptic axons. We reasoned that if the inhibitory component of the response results from a single all or nothing event, for example if the excitatory input triggers a single distal Ca^2+^ spike followed by a substantial afterhyperpolarization (Llinás & Sugimori, 1980) or a single filtered action potential propagating from an electrically connected neurone (Mann‐Metzer & Yarom, 1999; Nolan *et al*. 1999), then its amplitude should be independent of stimulus intensity. In contrast, we found that the mean amplitude of the synaptically evoked excitation and inhibition both vary as a function of light intensity (Fig. 1 *G* and *H*), while the ratio of the amplitudes of the excitatory and inhibitory components was independent of stimulus intensity (Fig. 1 *I*), suggesting that activation of additional axons similarly recruits both components of the PSP. These observations are consistent with models of IO neurones in which excitatory inputs at multiple distinct synaptic locations generate local, biphasic electrogenic responses that sum with one another at the soma (Kistler & De Zeeuw, 2005).

### The motor cortex makes functional monosynaptic connections with the IO

Because inputs activated optically in Thy1‐ChR2 mice could in principle arise from many different brain regions, we wanted to also investigate inputs from a single defined brain area. Therefore, in a second approach we asked if axons originating from neurones in the motor cortex establish functional synaptic connections with IO neurones, and whether their properties are similar to responses activated in Thy1‐ChR2 mice. This input is of interest as a potential pathway for interactions between the neocortex and cerebellum (Thach, 2007; Watson *et al*. 2009). However, while electrical activation of the motor cortex generates PSPs in IO neurones (Crill, 1970) and anatomical evidence indicates that axons from motor cortex have collaterals that pass through the IO (Saint‐Cyr, 1983), it is not clear whether these data reflect a functional monosynaptic projection to the IO or how activation of axons from the motor cortex impacts signalling by IO neurones (Watson *et al*. 2009).

**Figure 2:**
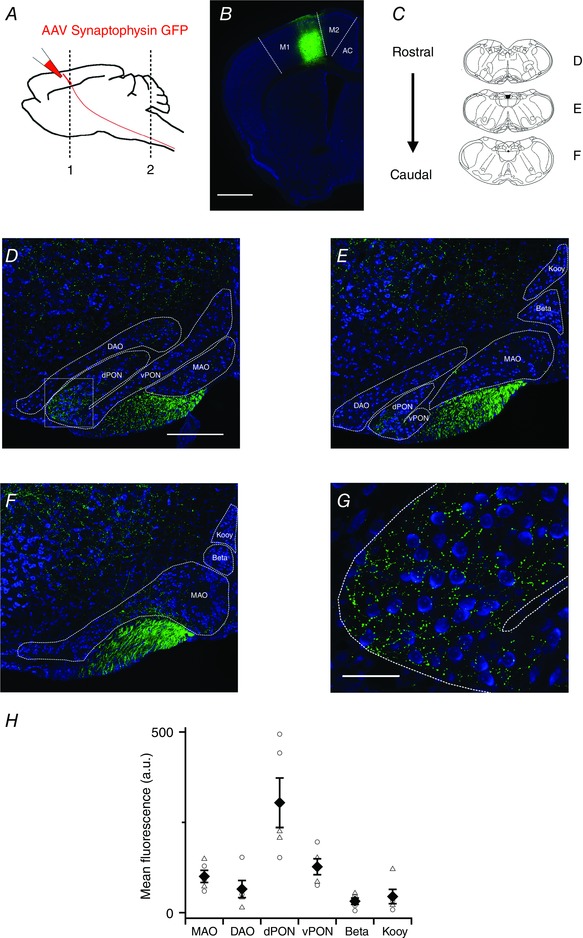
Projections from motor cortex differentially target IO nuclei *A*, strategy for injection of synaptophysin‐eGFP to label cortico‐olivary projections. *B*, coronal section showing eGFP expression (green) following a viral injection targeting M1. Scale bar 1000 μm. *C*, schematic diagram highlighting coronal sections used in *D–F*. *D–F*, coronal sections showing synaptophysin‐eGFP expression in the IO. Scale bar 200 μm. Abbreviations are: medial accessory olive (MAO), dorsal accessory olive (DAO), dorsal principal olivary nucleus (dPON), ventral principal olivary nucleus (vPON), beta nucleus (Beta) and the dorsal cap of Kooy (Kooy). *G*, high magnification image of the dashed area highlighted in *D*. Scale bar 50 μm. *H*, pooled data showing mean fluorescence intensity in each nucleus. Filled diamonds indicate the population mean (±SEM). Open circles are data points from mice with eGFP restricted to M1 and open triangles are data points from mice in which eGFP labelling was also present in adjacent more medial cortical structures. Abbreviation: arbitrary units (a.u.).

When we used an adeno‐associated virus (AAV) to express the presynaptic protein synaptophysin conjugated to eGFP (AAV‐synaptophysin‐eGFP) in neurones in the motor cortex, we found labelled axons in the IO (Fig. 2). In three injections eGFP labelling was restricted to M1 (Fig. 2 *B*) and in a further two injections labelling extended to adjacent M2 and anterior cingulate cortex (not shown). Evaluation of labelling across the rostro‐caudal extent of the IO (Fig. 2 *C*) revealed eGFP labelling in several nuclei of the IO (Fig. 2 *C–E*). Terminal labelling was largely in the region between cell bodies indicating that the targets of the motor cortex axons are extra‐somatic (Fig. 2 *F*). Quantification of fluorescence intensity indicated differences in labelling between nuclei (*F*_5,24_ = 9.34, *P* = 4.8 × 10^−5^, one‐way ANOVA, *N* = 5), with the strongest signal in the dorsal principal olivary nucleus (PON) (Fig. 2 *F* and *H*). We also observed moderate labelling in the medial accessory nucleus and ventral PON (Fig. 2 *H*). The fluorescence intensity was greatest in the PON when injections were restricted to M1 (Fig. 2 *H*). Together, these data suggest a robust projection from M1 that appears to preferentially target extra‐somatic regions of neurones in the dorsal PON.

**Figure 3:**
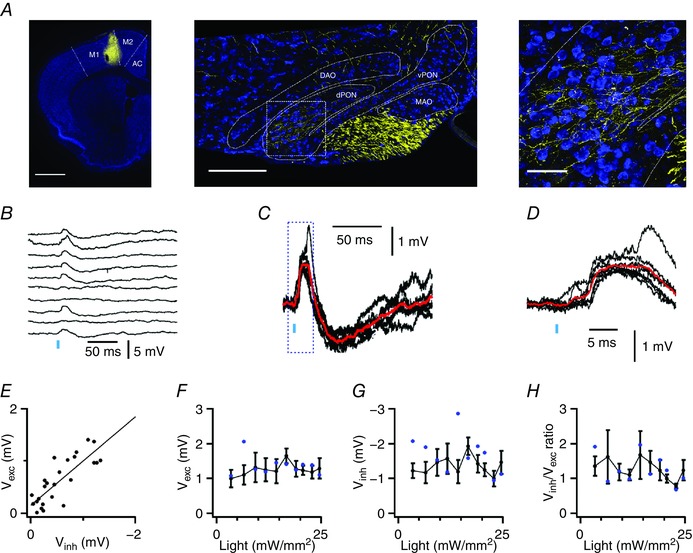
Bidirectional long‐range synaptic input to IO neurones *A*, coronal sections illustrate AAV‐ChR2‐Venus signal (yellow) in the motor cortex (left), in corticospinal tracts and principal olivary nucleus of the IO (middle), and in the IO at higher magnification (right). Scale bars are 1 mm (left), 200 μm (centre) and 50 μm (right). *B*, example responses of an IO neurone to optical activation of inputs from the motor cortex. *C* and *D*, traces from *B* overlaid and at a higher gain (*C*) and on a faster time scale (*D*). Average responses are in red. *E*, amplitude of individual inhibitory components are plotted as a function of the amplitude of the excitatory component for the recording in *G*. The line indicates linear regression (*R* ^2^ = 0.29, *P* = 2.4 × 10^−4^). *F–H*, *V*_exc_ (*F*), *V*_inh_ (*G*) and *V*_inh_/*V*_exc_ (*H*) plotted as a function of stimulus intensity (*n* = 7 cells). Data from the neurone in *B–E* are shown as blue circles.

To investigate functional properties of projections from the motor cortex to the IO we used AAV expressing ChR2 and Venus (AAV‐ChR2‐Venus). Following injection of AAV‐ChR2‐Venus into the motor cortex, we again observed labelling in the dorsal PON (Fig. 3 *A*). In contrast to punctate labelling observed following injection of AAV‐synaptophysin‐eGFP, continuous sections of axon were labelled following injection of AAV‐ChR2‐venus (Fig. 3 *A*). We activated ChR2‐expressing axons while recording the membrane potential of neurones in the PON (Fig. 3 *B*). The resulting PSPs were again bidirectional (*n* = 8 of 8 neurones responding to maximal intensity of stimulation) (Fig. 3 *B–D*). Responses had short latencies (2.37 ± 0.26 ms, *n* = 7, *N* = 7), which showed little variability (SD = 1.02 ± 0.17 ms, *n* = 7, *N* = 7) indicating that they are monosynaptic. Just as for responses recorded in Thy1‐ChR2 mice, the amplitude of the excitatory and inhibitory components were closely correlated indicating that both components have a common axonal origin (mean *R* ^2^ = 0.22 ± 0.05, *P* < 0.08, range *R* ^2^ = 0.08–0.45 and *P* = 0.08–0.0002, *n* = 7, *N* = 7) (Fig. 3 *E*). However, in contrast to responses of IO neurones from Thy1‐ChR2 mice, failures were visible on 29 ± 0.05% of trials using a maximal stimulus intensity (e.g. Fig. 3 *B*), and response amplitudes were relatively small and insensitive to changes in light intensity (Fig. 3 *F*), suggesting that they result from activation of relatively few axons. Together, these data indicate that IO neurones receive direct synaptic inputs from neurones in the neocortex and activation of these projections generates bidirectional PSPs.

### Depolarizing and hyperpolarizing components of bidirectional responses require activation of ionotropic glutamate receptors

What synaptic mechanisms mediate the bidirectional responses of IO neurones to synaptic input? In many brain areas bidirectional synaptic responses reflect initial direct excitation followed by slower recruitment of feed‐forward or feed‐back inhibition meditated by GABAergic interneurones (Isaacson & Scanziani, 2011). To test this possibility we examined the effect of blocking GABA_A_ receptors with picrotoxin. In this and subsequent experiments, unless indicated otherwise, we focus on responses to neocortical axons activated by maximal light intensity, and on responses evoked in neurones from Thy1‐ChR2 mice with light intensities adjusted to generate excitatory components with mean amplitude between 1 and 3 mV.

**Figure 4:**
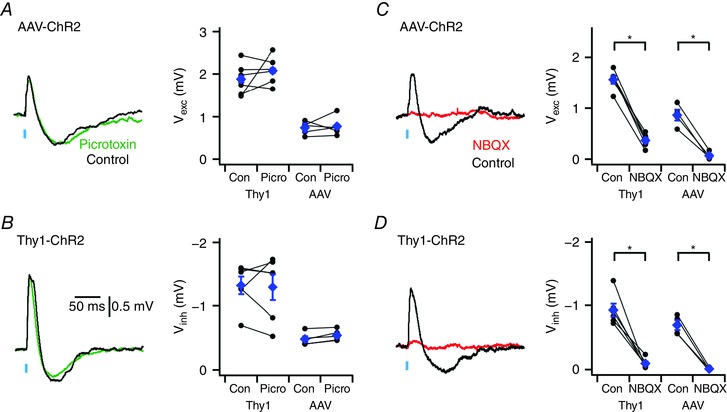
Depolarizing and hyperpolarizing components of synaptic responses are mediated by glutamate receptors *A* and *B*, examples (left) of synaptic responses to optical activation of neocortical inputs (*A*) and inputs in Thy1‐ChR2‐YFP mice (*B*) before (black traces) and during block of GABA_A_ receptors with picrotoxin (green traces). Population data (right) indicate that picrotoxin does not modify excitatory (*P* = 0.89 and *P* = 0.32, for AAV (*n* = 5, *N* = 5) and Thy1‐ChR2‐YFP (*n* = 6, *N* = 6) experiments, respectively, paired *t* test) or inhibitory components (*P* = 0.57 and *P* = 0.86, paired *t* test). *C* and *D*, examples (left) of synaptic responses to optical stimulation of motor cortex neurones expressing AAV‐ChR2‐Venus (*C*) and of inputs in Thy1‐ChR2 mice (*D*) before and during block of GluA receptors with NBQX. Population data (right) indicate that NBQX abolishes excitatory (*P* = 0.017, *n* = 4, *N* = 4 and *P* = 9.5 × 10^−6^, *n* = 6, *N* = 6, paired *t* test) and inhibitory components (*P* = 0.033, *n* = 4, *N* = 4 and *P* = 8.6 × 10^−4^, *n* = 6, *N* = 6, paired *t* test). Data are presented as means ± SEM. ^*^ *P* < 0.05 control *vs*. NBQX (paired *t* test).

While IO neurones are known to respond to GABA_A_‐mediated synaptic inputs (Best & Regehr, 2009), we found no effect of the GABA_A_ receptor antagonist picrotoxin on responses to optical activation of neocortical axons (*P* = 0.89 and 0.57 for excitatory and inhibitory components, respectively, *n* = 5, *N* = 5, paired *t* test) (Fig. 4 *A*) or on responses to inputs in Thy1‐ChR2 mice (*P* = 0.32 and 0.86, *n* = 6, *N* = 6, paired *t* test) (Fig. 4 *B*). In contrast, we found that the GluA receptor antagonist NBQX abolished excitatory and inhibitory components of responses to neocortical inputs (*P* = 2.3 × 10^−3^ and 2.7 × 10^−3^, respectively for each component, *n* = 4, *N* = 4 paired *t* test) (Fig. 4 *C*) and to activation of inputs in Thy1‐ChR2 mice (*P* = 9.5 × 10^−6^ and 8.6 × 10^−4^, *n* = 6, *N* = 6, paired *t* test) (Fig. 4 *D*). Thus, GluA receptors are required for the bidirectional synaptic responses of IO neurones to long‐range inputs. Together, with the insensitivity of PSPs to block of GABA receptors and the close correlation between their excitatory and inhibitory components (Figs 1 *F* and 3 *E*), these data indicate that both components of the PSP are generated by synaptic activation of GluAs on IO neurones.

### Hyperpolarizing components require calcium‐activated potassium channels and suppress responses to high‐frequency input

**Figure 5:**
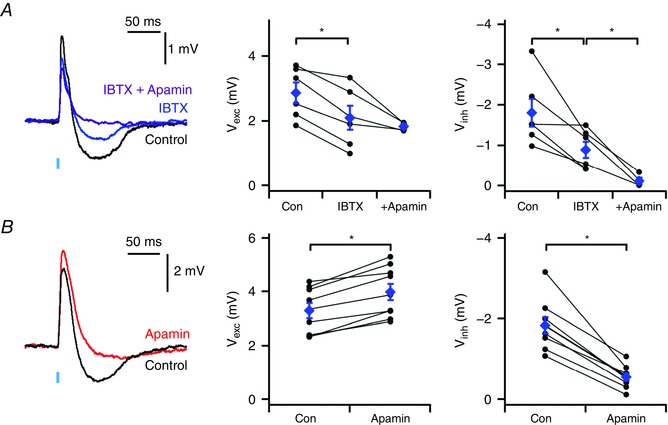
Bidirectional responses require calcium‐activated potassium channels *A*, examples (left) of synaptic responses of neurones from Thy1‐ChR2‐YFP mice to optical activation of inputs before and during block of BK channels with iberiotoxin (IBTX), and subsequent block with iberiotoxin and apamin. Population data (centre and right) indicate that iberiotoxin reduces the inhibitory component (*V*_inh_: *P* = 0.046, *n* = 6, *N* = 6, paired *t* test) and that subsequent addition of apamin blocks the inhibitory component (*V*_inh_: *P* = 0.006, *n* = 4, *N* = 4, paired *t* test). *B*, examples of synaptic responses to optical activation of inputs in Thy1‐ChR2‐YFP before and during block of SK channels with apamin. Population data (centre and right) indicate that apamin blocks the inhibitory component (*P* = 2.6 × 10^−5^, *n* = 9, *N* = 8, paired *t* test) while increasing the amplitude of the excitatory component (*P* = 7.3 × 10^−5^, *n* = 9, *N* = 8, paired *t* test). Data are presented as means ± SEM. ^*^ *P* < 0.05 (paired *t* test).

We reasoned that a possible mechanism for generation of the inhibitory component of the GluA synaptic response would be through recruitment of calcium‐activated potassium channels following dendritic calcium influx via high‐voltage activated (HVA) channels (cf. Kistler & De Zeeuw, 2005). To test this we examined the effects of blocking large‐conductance (BK) or small‐conductance (SK) calcium‐activated potassium channels on responses to inputs in Thy1‐ChR2 mice. The selective BK blocker iberiotoxin (100 nm) reduced the inhibitory component of the PSP (*V*_exc_, *P* = 0.14; *V*_inh_, *P* = 0.046, *n* = 6, *N* = 6, paired *t* test) (Fig. 5 *A*). Subsequent application of the SK‐specific blocker apamin (200 nm) completely abolished the inhibitory component (*P* = 0.006, *n* = 4, *N* = 4, paired *t* test) and reduced the excitatory component (*P* = 0.007, *n* = 4, *N* = 4, paired *t* test). In separate experiments SK blockade with apamin alone had little effect upon the excitatory component (*P* = 2.6 × 10^−5^, *n* = 9, *N* = 8, paired *t* test), but greatly reduced the inhibitory response, leaving only a small residual component (Fig. 5 *B*, *P* = 7.3 × 10^−5^, *n* = 9, *N* = 8, paired *t* test). Thus, recruitment of small conductance calcium‐activated potassium channels is required for the inhibitory, but not for the excitatory component of the response of IO neurones to glutamatergic synaptic input.

**Figure 6:**
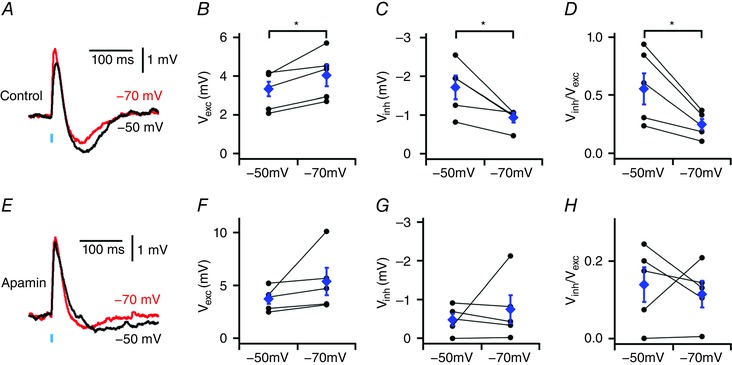
Voltage dependence of inhibitory and excitatory components of PSPs *A*, examples of PSPs evoked in Thy1‐ChR2‐YFP mice when the membrane potential is adjusted to −50 mV or −70 mV. *B–D*, effect of adjusting the membrane potential on the amplitude of the excitatory component (*V*_exc_: *P* = 0.020, *n* = 5, *N* = 5, paired *t* test) (*B*), the inhibitory component (*V*_inh_: *P* = 0.028, *n* = 5, *N* = 5, paired *t* test) (*C*) and the ratio between the amplitudes of the inhibitory and excitatory components (*V*_inh_/*V*_exc_: *P* = 0.022, *n* = 5, *N* = 5, paired *t* test) (*D*). *E–H*, as for *A–D*, but during application of apamin (*V*_exc_: *P* = 0.169, *V*_inh:_ *P* = 0.537, *V*_inh_/*V*_exc_: *P* = 0.673, *n* = 5, *N* = 5, paired *t* test). Data are presented as means ± SEM. ^*^ *P* < 0.05 control *vs*. apamin (paired *t* test).

If the inhibitory component of the PSP is mediated by activation of calcium‐activated potassium channels then we expect it to be sensitive to membrane potential hyperpolarization. Consistent with this, injection of negative current to hyperpolarize the membrane potential, from −50 mV to −70 mV, increased the amplitude of the excitatory component (*P* = 0.020, *n* = 5, *N* = 5, paired *t* test) of the PSP, reduced the amplitude of the inhibitory component (*P* = 0.028, *n* = 5, *N* = 5, paired *t* test), and reduced the ratio of the inhibitory to excitatory component amplitudes (*P* = 0.022, *n* = 5, *N* = 5, paired *t* test) (Fig. 6 *A*). In the presence of apamin the small residual inhibitory component was no longer dependent on membrane potential (*P* = 0.537, *n* = 5, *N* = 5, paired *t* test) (Fig. 6 *B*).

**Figure 7:**
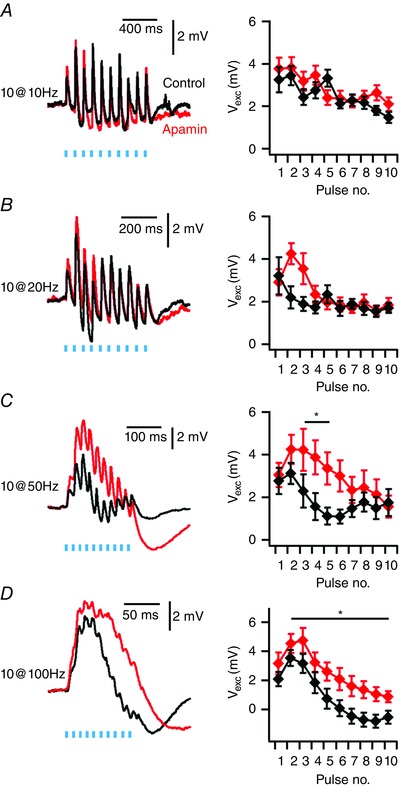
Temporal summation is enhanced by blocking the apamin‐sensitive component of postsynaptic responses *A–D*, examples of responses in Thy1‐ChR2‐YFP mice to trains of 10 stimuli delivered at 10 Hz (*A*), 20 Hz (*B*), 50 Hz (*C*) and 100 Hz (*D*) (left) and mean values of peak depolarization following each stimulus (right). Peak depolarization following each stimulus (*V*_exc_) during 50 and 100 Hz trains is increased by apamin (50 Hz: Condition *F*_1,80_ = 4.736, *P* = 0.033; 100 Hz: Condition *F*_1,80_ = 8.349, *P* = 0.005, *n* = 6, *N* = 6, two‐way ANOVA), but not at 10 and 20 Hz (10 Hz: Condition *F*_1,80_ = 0.047, *P* = 0.829; 20 Hz: Condition *F*_1,80_ = 0.047, *P* = 0.83, *n* = 6, *N* = 6, two‐way ANOVA). Data are presented as means ± SEM. ^*^ *P* < 0.05 control *vs*. apamin (Holm–Bonferroni *post hoc* comparison).

Finally, we wanted to know if the inhibitory component of the synaptic response influences temporal integration of synaptic inputs to IO neurones. To test this we examined responses to activation of inputs in Thy1‐ChR2 mice by 10 consecutive stimuli delivered at frequencies of either 10, 20, 50 or 100 Hz (*n* = 6 neurones from *N* = 6 mice) (Fig. 7). In agreement with previous observations (Turecek *et al*. 2014), we found that synaptic responses were depressed during trains of high‐frequency stimulation. We investigated the role of the inhibitory component of the synaptic response by blocking SK channels with apamin. During stimulation at 10–20 Hz the stimulation interval is similar to the duration of the inhibitory component of responses to isolated stimuli, and apamin had no effect on summation of the excitatory component of the synaptic responses (*P* > 0.8, *n* = 6, *N* = 6, two‐way ANOVA) (Fig. 7 *A*). In contrast, during stimulation at higher frequencies (inter‐stimulus interval < 20 ms) the peak depolarization in response to each stimulus was increased in the presence of apamin (50 Hz: *P* = 0.033, 100 Hz: *P* = 0.005, *n* = 6, *N* = 6, two‐way ANOVA) (Fig. 7 *B–D*). This is consistent with the apamin‐sensitive hyperpolarizing component of each PSP reducing the peak depolarization obtained during a following PSP. High frequency stimulation also revealed an additional slow inhibitory response that appeared to be increased in amplitude in the presence of apamin. This slow inhibitory component is most clearly seen as a large hyperpolarization at the end of a train of 50 Hz stimuli (Fig. 7 *C*).

## Discussion

Excitation followed by delayed inhibition is a feature of synaptic activity in many neuronal circuits (Isaacson & Scanziani, 2011). We demonstrate that responses of IO neurones to long‐range inputs have a similar biphasic organization. However, whereas in other brain areas delayed inhibition is mediated by interneurones, we find that in the IO it results from intrinsic electrical signalling downstream of GluA activation. Inhibitory components of synaptic responses in IO neurones can be activated by very few axons, while recruitment of additional axons generates hyperpolarizing responses that scale linearly with the amplitude of the preceding depolarization. The inhibitory component requires calcium‐activated potassium channels and is observed following activation of inputs from the motor cortex and more general activation of axons expressing ChR2 in Thy1‐ChR2 mice. Activation of the apamin‐sensitive inhibitory component opposes temporal summation of inputs active at intervals ≤ 20 ms, suggesting an important role for active conductances in synaptic integration within the IO.

### Projections from the motor cortex target specific nuclei within the IO

Interactions between the motor cortex and olivo‐cerebellar system are important for control of movement (Middleton & Strick, 2000). While previous anatomical evidence suggested that axons from the motor cortex reach the IO (Sousa‐Pinto, 1969; Saint‐Cyr, 1983), it was not clear whether these axons make functional connections, or how postsynaptic neurones respond to their activation. We find that axons from the motor cortex make functional synaptic connections onto principal neurones in the inferior olive. The highest density of terminal labelling was in the dorsal PON and was observed when injections were focused on M1. More medial injections that included a smaller region of M1, along with M2 or cingulate cortex, also labelled terminals in the IO, but their density was reduced, suggesting that projections arise primarily from M1. Nevertheless, further investigation will be required to establish whether or not other neocortical structures also project to the IO. Because our injections focused on more medial parts of M1, it also remains to be determined whether projections originate from all of M1, whether the projections follow a topographical organization and if so whether this topography is consistent with previous evidence for topographical organization of olivo‐cerebellar modules (Apps & Garwicz, 2005). By expressing ChR2 in neurones projecting from the motor cortex we were able to investigate their functional impact in the IO. We show that neurones in the IO generate bidirectional responses to glutamatergic inputs from the motor cortex, with late hyperpolarizing components requiring apamin‐sensitive calcium‐activated potassium channels. The bidirectional character of responses to motor cortex input suggests that the timing of action potentials in the motor cortex may be critical to their impact on the IO.

### Mechanisms for synaptic integration in the IO

Our results corroborate several predictions of a two‐stage theoretical model of synaptic integration in the IO (Kistler & De Zeeuw, 2005). A central prediction of this model is that in a first stage of integration excitatory inputs initiate local spikes within dendritic spines. Because these spikes are followed by an afterhyperpolarization, they are predicted to propagate to the soma as bidirectional potentials (Kistler & De Zeeuw, 2005). Our findings that GluA responses have slow inhibitory components (Figs 1 *D* and 3 *C*), that excitatory and inhibitory components result from activation of the same presynaptic axons (Figs 1 *F* and 3 *E*), and that the inhibitory component requires SK channels, are consistent with this prediction. The presence of a second component of the initial excitatory response (Figs 1 *E* and 3 *D*) is also consistent with delayed activation of a distal spike. A second key prediction of the two‐stage model is that if biphasic responses generated in different glomeruli are integrated at the soma they will sum independently (Kistler & De Zeeuw, 2005). Consistent with this, recruitment of additional presynaptic axons causes both inhibitory and excitatory components to increase in parallel (Figs 1 *G* and 3 *F*), while the relative amplitude of the depolarizing and hyperpolarizing responses appears constant in individual neurones (Fig. 3 *E* and *J*).

Previous investigations have focused on mechanisms for generation of sinusoidal oscillatory activity by neurones in the IO (e.g. Benardo & Foster, 1986; Llinás & Yarom, 1986). Models of sinusoidal oscillatory activity incorporate calcium‐activated potassium channels that our data suggest also play roles in generation of bidirectional synaptic responses (e.g. Schweighofer *et al*. 1999; De Gruijl *et al*. 2012). This raises the question of the relationship between these phenomena. In this respect, it is striking that in our *in vitro* conditions and in previous *in vivo* experiments less than 30% of IO neurones were reported to demonstrate spontaneous sinusoidal oscillations (Khosrovani *et al*. 2007). In contrast, all of the IO neurones that we recorded here demonstrate bidirectional responses to glutamatergic synaptic impact. The waveform of the synaptic responses that we observe differs from sinusoidal oscillations and resembles more closely low‐threshold Ca^2+^ oscillations also recorded from IO neurones *in vivo* (Khosrovani *et al*. 2007). Bidirectional synaptic responses also differ from sinusoidal oscillations in that they are discrete events and are not accompanied by reverberant oscillatory activity (cf. Benardo & Foster, 1986; Llinás & Yarom, 1986). The absence of reverberant activity also distinguishes bidirectional synaptic responses from the apamin‐sensitive afterhyperpolarization (AHP) (Bal & McCormick, 1997; Khosrovani *et al*. 2007). Indeed, while the apamin‐sensitive AHP has a fixed amplitude, the peaks of bidirectional responses vary continuously (from <1 mV to >5 mV) as a function of the number of activated inputs. Interestingly, whereas sinusoidal oscillations and the apamin‐sensitive AHP can be accounted for by models of interactions between somatic and dendritic ion channels (Schweighofer *et al*. 1999; De Gruijl *et al*. 2012), the properties of bidirectional synaptic responses that we observed experimentally are predicted by models incorporating integration by active dendritic spines (Kistler & De Zeeuw, 2005). These models and our data are consistent with evidence from other brain areas for localization of SK channels to dendritic spines (Faber *et al*. 2005; Ngo‐Anh *et al*. 2005). The small amplitude of minimal bidirectional responses is also consistent with highly localized activation (cf. Fig. 1 and Kistler & De Zeeuw, 2005). It will therefore be an important question for future studies to address whether bidirectional synaptic responses and sinusoidal membrane potential oscillations are mediated by ion channels at different subcellular locations. Given the prevalence of gap junctions between dendrites of neurones in the IO (Sotelo *et al*. 1974), it will also be of interest to establish the impact of electrical coupling on integration of bidirectional responses to glutamatergic inputs.

### Functional implications of bidirectional responses of IO neurones to glutamatergic inputs

While the IO is a critical integrative centre for cerebellar‐dependent behaviours, the nature of the computation that it performs is not clear. The IO conveys information about the timing of stimuli to the cerebellar cortex (De Zeeuw *et al*. 1998), signals reach the IO through glutamatergic synapses (Lang, 2001, 2002) and glutamatergic inputs to the IO are required for cerebellar behaviours (Carrel *et al*. 2013). Our results suggest that the complex active signalling properties of the IO implement unique rules for integration of glutamatergic synaptic input by the IO (Kistler & De Zeeuw, 2005). In particular, the apamin‐sensitive inhibitory component of PSPs that we identify here appears to oppose summation of inputs activated at intervals ≤ 20 ms. Thus, subthreshold integration will tend to privilege stimuli that arrive in temporally restricted windows and will potentially increase the temporal contrast of excitatory stimuli, limiting the efficacy of synaptic integration of late‐coming stimuli. These functions complement the view that a primary function of the IO is as a generator of synchronized oscillatory states (Llinás, 1988). In particular, the integrative mechanisms that we describe here may enable climbing fibre signals to encode the onset of stimuli of importance for coordinated movement.

## Additional information

### Competing interests

None declared.

### Author contributions

All experiments were performed in the Centre for Integrative Physiology at the University of Edinburgh. All authors contributed to: conception and design of the experiments; acquisition, analysis and interpretation of data; and drafting and revising the manuscript for important intellectual content. All authors approved the final version of the manuscript and agree to be accountable for all aspects of the work in ensuring that questions related to the accuracy or integrity of any part of the work are appropriately investigated and resolved. All persons designated as authors qualify for authorship, and all those who qualify for authorship are listed.

### Funding

This work was supported by the Medical Research Council (G0501216), the Wellcome Trust (093295/Z/10/Z) and the BBSRC (Bb/H020284/1).

## References

[tjp12072-bib-0001] Apps R & Garwicz M (2005). Anatomical and physiological foundations of cerebellar information processing. Nat Rev Neurosci 6, 297–311. 15803161 10.1038/nrn1646

[tjp12072-bib-0002] Arenkiel BR , Peca J , Davison IG , Feliciano C , Deisseroth K , Augustine GJ , Ehlers MD & Feng G (2007). In vivo light‐induced activation of neural circuitry in transgenic mice expressing channelrhodopsin‐2. Neuron 54, 205–218. 17442243 10.1016/j.neuron.2007.03.005 PMC3634585

[tjp12072-bib-0003] Armstrong DM (1974). Functional significance of connections of the inferior olive. Physiol Rev 54, 358–417. 4362162 10.1152/physrev.1974.54.2.358

[tjp12072-bib-0004] Badura A , Schonewille M , Voges K , Galliano E , Renier N , Gao Z , Witter L , Hoebeek FE , Chedotal A & De Zeeuw CI (2013). Climbing fiber input shapes reciprocity of Purkinje cell firing. Neuron 78, 700–713. 23643935 10.1016/j.neuron.2013.03.018

[tjp12072-bib-0005] Bal T & McCormick DA (1997). Synchronized oscillations in the inferior olive are controlled by the hyperpolarization‐activated cation current *I* _h_ . J Neurophysiol 77, 3145–3156. 9212264 10.1152/jn.1997.77.6.3145

[tjp12072-bib-0006] Bazzigaluppi P , Ruigrok T , Saisan P , De Zeeuw CI & de Jeu M (2012). Properties of the nucleo‐olivary pathway: an in vivo whole‐cell patch clamp study. PLoS One 7, e46360. 23029495 10.1371/journal.pone.0046360 PMC3459892

[tjp12072-bib-0007] Benardo LS & Foster RE (1986). Oscillatory behavior in inferior olive neurons: mechanism, modulation, cell aggregates. Brain Res Bull 17, 773–784. 3026580 10.1016/0361-9230(86)90089-4

[tjp12072-bib-0008] Best AR & Regehr WG (2009). Inhibitory regulation of electrically coupled neurons in the inferior olive is mediated by asynchronous release of GABA. Neuron 62, 555–565. 19477156 10.1016/j.neuron.2009.04.018 PMC3261724

[tjp12072-bib-0009] Carrel AJ , Zenitsky GD & Bracha V (2013). Blocking glutamate‐mediated inferior olivary signals abolishes expression of conditioned eyeblinks but does not prevent their acquisition. J Neurosci 33, 9097–9103. 23699520 10.1523/JNEUROSCI.3129-12.2013 PMC3865544

[tjp12072-bib-0010] Crill WE (1970). Unitary multiple‐spiked responses in cat inferior olive nucleus. J Neurophysiol 33, 199–209. 4313283 10.1152/jn.1970.33.2.199

[tjp12072-bib-0011] De Gruijl JR , Bazzigaluppi P , de Jeu MT & De Zeeuw CI (2012). Climbing fiber burst size and olivary sub‐threshold oscillations in a network setting. PLoS Comput Biol 8, e1002814. 23271962 10.1371/journal.pcbi.1002814 PMC3521668

[tjp12072-bib-0012] De Zeeuw CI , Chorev E , Devor A , Manor Y , Van Der Giessen RS , De Jeu MT , Hoogenraad CC , Bijman J , Ruigrok TJ , French P , Jaarsma D , Kistler WM , Meier C , Petrasch‐Parwez E , Dermietzel R , Sohl G , Gueldenagel M , Willecke K & Yarom Y (2003). Deformation of network connectivity in the inferior olive of connexin 36‐deficient mice is compensated by morphological and electrophysiological changes at the single neuron level. J Neurosci 23, 4700–4711. 12805309 10.1523/JNEUROSCI.23-11-04700.2003 PMC6740782

[tjp12072-bib-0013] De Zeeuw CI , Hoebeek FE , Bosman LW , Schonewille M , Witter L & Koekkoek SK (2011). Spatiotemporal firing patterns in the cerebellum. Nat Rev Neurosci 12, 327–344. 21544091 10.1038/nrn3011

[tjp12072-bib-0014] De Zeeuw CI , Simpson JI , Hoogenraad CC , Galjart N , Koekkoek SK & Ruigrok TJ (1998). Microcircuitry and function of the inferior olive. Trends Neurosci 21, 391–400. 9735947 10.1016/s0166-2236(98)01310-1

[tjp12072-bib-0015] Faber ES , Delaney AJ & Sah P (2005). SK channels regulate excitatory synaptic transmission and plasticity in the lateral amygdala. Nat Neurosci 8, 635–641. 15852010 10.1038/nn1450

[tjp12072-bib-0016] Groh A , de Kock CP , Wimmer VC , Sakmann B & Kuner T (2008). Driver or coincidence detector: modal switch of a corticothalamic giant synapse controlled by spontaneous activity and short‐term depression. J Neurosci 28, 9652–9663. 18815251 10.1523/JNEUROSCI.1554-08.2008 PMC6671213

[tjp12072-bib-0017] Isaacson JS & Scanziani M (2011). How inhibition shapes cortical activity. Neuron 72, 231–243. 22017986 10.1016/j.neuron.2011.09.027 PMC3236361

[tjp12072-bib-0018] Khosrovani S , Van Der Giessen RS , De Zeeuw CI & De Jeu MT (2007). In vivo mouse inferior olive neurons exhibit heterogeneous subthreshold oscillations and spiking patterns. Proc Natl Acad Sci USA 104, 15911–15916. 17895389 10.1073/pnas.0702727104 PMC2000380

[tjp12072-bib-0019] Kistler WM & De Zeeuw CI (2005). Gap junctions synchronize synaptic input rather than spike output of olivary neurons. Prog Brain Res 148, 189–197. 15661191 10.1016/S0079-6123(04)48016-9

[tjp12072-bib-0020] Lang EJ (2001). Organization of olivocerebellar activity in the absence of excitatory glutamatergic input. J Neurosci 21, 1663–1675. 11222657 10.1523/JNEUROSCI.21-05-01663.2001 PMC6762933

[tjp12072-bib-0021] Lang EJ (2002). GABAergic and glutamatergic modulation of spontaneous and motor‐cortex‐evoked complex spike activity. J Neurophysiol 87, 1993–2008. 11929918 10.1152/jn.00477.2001

[tjp12072-bib-0022] Lefler Y , Yarom Y & Uusisaari MY (2014). Cerebellar inhibitory input to the inferior olive decreases electrical coupling and blocks subthreshold oscillations. Neuron 81, 1389–1400. 24656256 10.1016/j.neuron.2014.02.032

[tjp12072-bib-0023] Llinás RR (1988). The intrinsic electrophysiological properties of mammalian neurons: insights into central nervous system function. Science 242, 1654–1664. 3059497 10.1126/science.3059497

[tjp12072-bib-0024] Llinás RR (2009). Inferior olive oscillation as the temporal basis for motricity and oscillatory reset as the basis for motor error correction. Neuroscience 162, 797–804. 19393291 10.1016/j.neuroscience.2009.04.045 PMC2861300

[tjp12072-bib-0025] Llinás R & Sugimori M (1980). Electrophysiological properties of *in vitro* Purkinje cell dendrites in mammalian cerebellar slices. J Physiol 305, 197–213. 7441553 10.1113/jphysiol.1980.sp013358 PMC1282967

[tjp12072-bib-0026] Llinás R & Yarom Y (1981). Electrophysiology of mammalian inferior olivary neurones *in vitro*. Different types of voltage‐dependent ionic conductances. J Physiol 315, 549–567. 6273544 10.1113/jphysiol.1981.sp013763 PMC1249398

[tjp12072-bib-0027] Llinás R & Yarom Y (1986). Oscillatory properties of guinea‐pig inferior olivary neurones and their pharmacological modulation: an *in vitro* study. J Physiol 376, 163–182. 3795074 10.1113/jphysiol.1986.sp016147 PMC1182792

[tjp12072-bib-0028] Long MA , Deans MR , Paul DL & Connors BW (2002). Rhythmicity without synchrony in the electrically uncoupled inferior olive. J Neurosci 22, 10898–10905. 12486184 10.1523/JNEUROSCI.22-24-10898.2002 PMC2834587

[tjp12072-bib-0029] Mann‐Metzer P & Yarom Y (1999). Electrotonic coupling interacts with intrinsic properties to generate synchronized activity in cerebellar networks of inhibitory interneurons. J Neurosci 19, 3298–3306. 10212289 10.1523/JNEUROSCI.19-09-03298.1999 PMC6782243

[tjp12072-bib-0030] Mathy A , Ho SS , Davie JT , Duguid IC , Clark BA & Hausser M (2009). Encoding of oscillations by axonal bursts in inferior olive neurons. Neuron 62, 388–399. 19447094 10.1016/j.neuron.2009.03.023 PMC2777250

[tjp12072-bib-0031] Middleton FA & Strick PL (2000). Basal ganglia and cerebellar loops: motor and cognitive circuits. Brain Res Brain Res Rev 31, 236–250. 10719151 10.1016/s0165-0173(99)00040-5

[tjp12072-bib-0032] Ngo‐Anh TJ , Bloodgood BL , Lin M , Sabatini BL , Maylie J & Adelman JP (2005). SK channels and NMDA receptors form a Ca^2+^‐mediated feedback loop in dendritic spines. Nat Neurosci 8, 642–649. 15852011 10.1038/nn1449

[tjp12072-bib-0033] Nolan MF , Logan SD & Spanswick D (1999). Electrophysiological properties of electrical synapses between rat sympathetic preganglionic neurones *in vitro* . J Physiol 519, 753–764. 10457088 10.1111/j.1469-7793.1999.0753n.x PMC2269542

[tjp12072-bib-0034] Ruigrok TJ & Voogd J (1995). Cerebellar influence on olivary excitability in the cat. Eur J Neurosci 7, 679–693. 7542527 10.1111/j.1460-9568.1995.tb00672.x

[tjp12072-bib-0035] Saint‐Cyr JA (1983). The projection from the motor cortex to the inferior olive in the cat. An experimental study using axonal transport techniques. Neuroscience 10, 667–684. 6196684 10.1016/0306-4522(83)90209-9

[tjp12072-bib-0036] Schindelin J , Arganda‐Carreras I , Frise E , Kaynig V , Longair M , Pietzsch T , Preibisch S , Rueden C , Saalfeld S , Schmid B , Tinevez JY , White DJ , Hartenstein V , Eliceiri K , Tomancak P & Cardona A (2012). Fiji: an open‐source platform for biological‐image analysis. Nat Methods 9, 676–682. 22743772 10.1038/nmeth.2019 PMC3855844

[tjp12072-bib-0037] Schweighofer N , Doya K & Kawato M (1999). Electrophysiological properties of inferior olive neurons: A compartmental model. J Neurophysiol 82, 804–817. 10444678 10.1152/jn.1999.82.2.804

[tjp12072-bib-0038] Sotelo C , Llinás R & Baker R (1974). Structural study of inferior olivary nucleus of the cat: morphological correlates of electrotonic coupling. J Neurophysiol 37, 541–559. 4827021 10.1152/jn.1974.37.3.541

[tjp12072-bib-0039] Sousa‐Pinto A (1969). Experimental anatomical demonstration of a cortico‐olivary projection from area 6 (supplementary motor area?) in the cat. Brain Res 16, 73–83. 5348863 10.1016/0006-8993(69)90086-9

[tjp12072-bib-0040] Sürmeli G , Marcu DC , McClure C , Garden DL , Pastoll H & Nolan MF (2015). Molecularly defined circuitry reveals input‐output segregation in deep layers of the medial entorhinal cortex. Neuron 88, 1040–1053. 26606996 10.1016/j.neuron.2015.10.041 PMC4675718

[tjp12072-bib-0041] Thach WT (2007). On the mechanism of cerebellar contributions to cognition. Cerebellum 6, 163–167. 17786811 10.1080/14734220701373530

[tjp12072-bib-0042] Turecek J , Yuen GS , Han VZ , Zeng XH , Bayer KU & Welsh JP (2014). NMDA receptor activation strengthens weak electrical coupling in mammalian brain. Neuron 81, 1375–1388. 24656255 10.1016/j.neuron.2014.01.024 PMC4266555

[tjp12072-bib-0043] Watson TC , Jones MW & Apps R (2009). Electrophysiological mapping of novel prefrontal–cerebellar pathways. Front Integr Neurosci 3, 18. 19738932 10.3389/neuro.07.018.2009 PMC2737490

